# Monochorea after acute contralateral pontine infarction: A case report

**DOI:** 10.1097/MD.0000000000032660

**Published:** 2023-01-20

**Authors:** Yun Su Hwang, Byoung-Soo Shin, Han Uk Ryu, Hyun Goo Kang

**Affiliations:** a Department of Neurology, Jeonbuk National University Medical School and Hospital, Jeonju, South Korea; b Research Institute of Clinical Medicine of Jeonbuk National University – Biomedical Research Institute of Jeonbuk National University Hospital, Jeonju, South Korea.

**Keywords:** monochorea, pontine infarction, post-stroke chorea

## Abstract

Chorea is a hyperkinetic movement characterized by random, brief, and involuntary muscle contractions. In stroke, a common cause of chorea, basal ganglia are anatomical locations that can cause chorea when a stroke occurs, and chorea is less frequently triggered by a stroke in other anatomical brain regions. Herein, we report a rare case of monochorea after acute contralateral pontine infarction.

**Patient concerns:**

A 32-year-old man visited the emergency room due to dysarthria and right hemiparesis that occurred approximately 6 hours and 30 minutes before the visit. A brain magnetic resonance image confirmed a diffusion restriction lesion in the left pons. The patient was initially diagnosed with acute infarction at the left pons and began to receive medical treatment with an antiplatelet agent and statin with admission.

**Diagnosis:**

Approximately 14 hours after the onset of the initial stroke symptoms, the patient complained of involuntary movement in the right arm for the first time. Intermittent, irregular involuntary movements were observed in the distal part of the right arm. This symptom was unpredictable and random, and a similar symptom was not observed in other parts of the patient’s body. Clinically, post-stroke monochorea was suspected.

**Interventions and outcomes:**

The symptom improved from day 5 without specific medical treatment for chorea.

**Lessons:**

The monochorea caused by the pontine lesion in this case was triggered by the direct lesions of the corticospinal tract, and its underlying pathophysiology remains unclear. However, abnormal movements can occur due to inadequate downstream activation or inhibition of the corticospinal tract, which is induced by functional abnormalities of the motor cortex. This case suggests that further investigation is needed on the mechanisms of direct corticospinal tract lesions for chorea.

## 1. Introduction

Chorea is a hyperkinetic movement characterized by random, brief, and involuntary muscle contractions.^[1]^ This symptom usually develops in the distal part of the body, and hemichorea is the most common type.^[2]^ In stroke – a common cause of chorea^[1]^ – the basal ganglia are anatomical locations that can cause chorea; specifically, the putamen is the main anatomical lesion causing chorea.^[1,3,4]^ Moreover, chorea is less frequently triggered by a stroke in other anatomical brain regions. Herein, we report a rare case of monochorea after acute contralateral pontine infarction.

## 2. Case presentation

A 32-year-old man visited our emergency room because of right hemiparesis beginning approximately 6 hours and 30 minutes prior. At presentation, the patient’s vital signs were within normal ranges, except for high blood pressure of 155/114 mm Hg. The patient had no specific medical history. Neurological examination revealed dysarthria, right facial palsy, and right hemiparesis, classified as approximately Medical Council Research grade 4.

**Figure 1.**
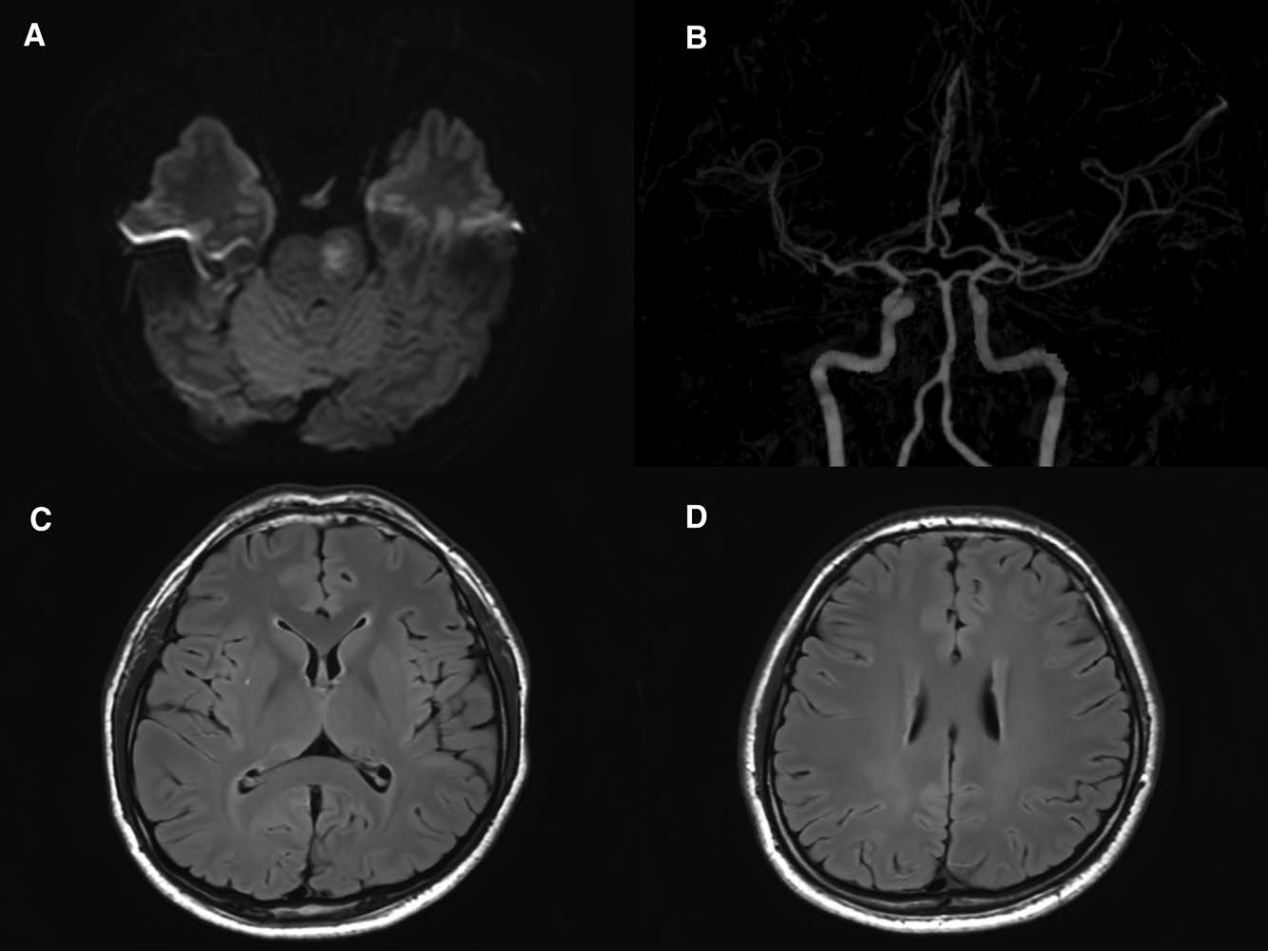
(A) Brain magnetic resonance image (MRI) reveals a diffusion restriction lesion in the left pons. (B) Brain computed tomography angiography shows no significant stenosis or occlusion in the major intracerebral arteries. (C and D) T2-fluid attenuated inversion recovery MRI reveals no other brain lesions in the basal ganglia, frontal cortex, and subcortex.

Brain magnetic resonance image confirmed a diffusion-restricted lesion in the left pons. Stenosis or occlusion of the intracranial main artery was not detected on brain computed tomography angiography; no other brain lesions were found (Fig. 1). Laboratory tests revealed an elevated low-density lipoprotein level of 173 mg/dL and a triglyceride level of 348 mg/dL. All other laboratory tests performed showed normal results. The patient was diagnosed with acute infarction in the left pons and hospitalized for medical treatment with antiplatelet agents. Approximately 14 hours after the onset of the hemiparesis, the patient complained of monochorea in the right arm. Intermittent and irregular involuntary movements were observed in the distal right arm. These movements were unpredictable and random and were not observed in other body parts (Supplementary Video, Supplemental Digital Content, http://links.lww.com/MD/I349). The chorea improved spontaneously on day 5 without specific medical treatment. The patient was discharged on the ninth hospital day and was followed up at the outpatient department. Currently, the patient exhibits no choreatic movements, although mild right hemiparesis remains.

## 3. Discussion

Chorea can be induced by diverse factors,^[5]^ and stroke is the most common cause of non-genetic chorea.^[1]^ Although chorea is a rare post-stroke complication, it is the most common post-stroke phenotype among abnormal movements.^[3]^ Although basal ganglia are the main anatomical locations for post-stroke chorea,^[6]^ lesions in the thalamus, subthalamic nucleus, caudate, globus pallidus interna, and contralateral cortex also can contribute to the occurrence of chorea.^[1,3]^ We have described a case of monochorea after acute pontine infarction, which is a rare anatomical location for the occurrence of chorea.^[1]^

One case of post-stroke pontine chorea has been reported; monochorea of the contralateral upper limb after acute pontine hemorrhage.^[3]^ However, that case differed from the current case, in which the pontine lesion invaded only the tegmentum, as the previous case initially presented with chorea, with the pontine hemorrhage confirmed subsequently; moreover, both the tegmentum and tectum were involved, and the lesion was larger than that in the present case. In summary, the key differences between this and the previous case were that chorea occurred with ischemia of only the tegmentum; moreover, a time difference existed in the onset of chorea. Thus, our case indicates that cerebral infarction can trigger symptoms later after onset, as the region related to chorea is gradually involved because cerebral infarction has the penumbra region along with the ischemic core, as such, the chorea may be improved as the penumbra region recovers. However, hemorrhage immediately causes cell death and vasogenic edema, resulting in chorea.

The development of hemichorea in lesions of the basal ganglia system is caused by increased motor cortical activation due to the dysfunction of the neuronal network connecting the basal ganglia and the frontal cortical motor areas.^[1]^ The monochorea caused by the pontine lesion in this and the previous case were triggered by direct lesions of the corticospinal tract, and its underlying pathophysiology remains unclear. Further, the reason why chorea manifested as monochorea only in the upper extremity and the mechanism was not elucidated. However, abnormal movements can occur due to inadequate downstream activation or inhibition of the corticospinal tract, which is induced by functional abnormalities of the motor cortex.^[4,6,7]^ Further, although we could not completely exclude other phenotypes of abnormal movements, we judged that the clinical features were consistent with chorea. Moreover, the gradual improvement and near-resolution after the acute stroke treatment also indicate post-stroke chorea.^[1]^

Therefore, despite insufficient evidence for chorea directly caused by corticospinal tract lesions, this case suggests that further investigation is needed to explore the mechanisms of direct corticospinal tract lesions for the occurrence of chorea.

## Author contributions

**Conceptualization:** Yun Su Hwang, Byoung-Soo Shin, Han Uk Ryu, Hyun Goo Kang.

**Data curation:** Yun Su Hwang, Han Uk Ryu.

**Formal analysis:** Yun Su Hwang, Byoung-Soo Shin, Hyun Goo Kang.

**Funding acquisition:** Hyun Goo Kang.

**Investigation:** Han Uk Ryu.

**Methodology:** Byoung-Soo Shin, Han Uk Ryu, Hyun Goo Kang.

**Supervision:** Byoung-Soo Shin, Han Uk Ryu, Hyun Goo Kang.

**Validation:** Byoung-Soo Shin.

**Visualization:** Yun Su Hwang, Hyun Goo Kang.

**Writing – original draft:** Yun Su Hwang, Hyun Goo Kang.

**Writing – review & editing:** Han Uk Ryu, Hyun Goo Kang.

## Supplementary Material
